# Analysis of *clasp2* Transcription Pattern in Male Germ Cells during Spermatogenesis: A Comparative Study in Zebrafish (*Danio rerio)* and Guppy (*Poecilia reticulata*)

**DOI:** 10.3390/ani13233617

**Published:** 2023-11-22

**Authors:** Serena Ricci, Maurizio Lazzari, Maria Gabriella Maurizii, Valeria Franceschini, Liliana Milani, Pietro Cacialli

**Affiliations:** Department of Biology, Geology and Environmental Sciences, University of Bologna, 40126 Bologna, Italy

**Keywords:** CLASP, microtubules, spermatogenesis, zebrafish, guppy

## Abstract

**Simple Summary:**

Fertility, in males and females, is linked to the correct formation of sexual line cells (spermatozoa and oocytes) through a physiological process called meiosis. In all vertebrates, meiosis is orchestrated by different molecular effectors, and among these, we focused our attention on CLASP2, a protein involved in cytoskeleton function. In several animal models, such as mice, flies, or frogs, this protein is known to participate in spermatozoa maturation. However, in fish, nothing was known about the presence of CLASP2 in sexual organs or whether it could have a role in spermatozoa formation. To investigate this point, we used two excellent vertebrate aquatic models: guppy and zebrafish. Our results open a window to knowledge about male sex cell formation effectors in fish. Considering the great affinity between zebrafish and humans, the study of CLASP2 in fish spermatogenesis could uncover a new target of male fertility in humans.

**Abstract:**

Cytoplasmic linker-associated protein-2 (CLASP2) is a member of the CLIP-associating proteins (CLASPs) family involved in the structure and function of microtubules and Golgi apparatus. Several studies performed using different mammalian and non-mammalian model organisms reported that CLASP2 controls microtubule dynamics and the organization of microtubule networks. In *Drosophila* and mice, an important role of CLASP2 during the development of germ cell lines has been uncovered. However, no study has clearly defined its role during fish germ cell differentiation. In the present study, we used two excellent aquatic animal models among teleost fish: zebrafish (*Danio rerio*) and guppy (*Poecilia reticulata*). Using qPCR, we found that the *clasp2* transcript level is significantly high in the testis of both fish. Then, by in situ hybridization, we localized the *clasp2* transcript in the spermatozoa of zebrafish and the spermatozeugmata of guppy. Our data suggest a potential role for this gene in the last stage of spermiogenesis in fish.

## 1. Introduction

Cytoplasmic linker-associated protein-2 (CLASP2) is one of the two mammalian conserved CLIP-associating proteins (CLASPs) involved in microtubule stabilization and acentrosomal nucleation [1,2,3]. In detail, CLASP2 strictly cooperates with its paralogue, CLASP1, to achieve functional mitotic spindle assembly [4]. In addition, several studies in epithelial [5,6] and mainly in neuronal [7,8] cell lines have defined a key role of CLASP2 in Golgi apparatus morphology, dynamics, and function. CLASP2 has received a lot of attention as a target in cell division aberration on the premise of carcinogenesis because of these important roles during cell division. *Clasp2* expression, indeed, was found to be upregulated in bladder cancer tissues, where it seems to promote endothelial-to-mesenchymal transition (EMT) and to be associated with tumor progression, gaining attention as a prognostic factor. Similarly, enrichment analysis on histological human specimens of lung cancer defined CLASP2 and its paralogue, CLASP1, as predictive markers of tumor recurrence [9,10,11].

The main studies that attempted to establish the role of CLASP2 in pathophysiological processes were reported in mice (*Mus musculus*). In particular, CLASP2 appears as a key factor during mouse neocortical development as it regulates neuron formation, migration, polarity, and synaptic function [7,12]. Interestingly, recent studies defined CLASP2 as a target “sensitive” to metabolic condition changes. Phospho-proteomic wide analysis of the brain from C57BL6 mice undergoing a high-fat diet showed a higher phosphorylation pattern of CLASP2 than tissues from mice on a chow diet. Further studies in vitro then clarified that CLASP2 undergoes insulin-stimulated phosphorylation, which has a crucial role in cytoskeleton modification and is necessary for GLUT4 transporter trafficking [13,14,15]. Also, CLASP2 was observed in mice to participate in microtubule destabilization, which is at the base of endothelial permeability and inflammation [16,17].

Although a wide body of literature is present about CLASP2 roles in mammalian cell pathways, the first evidence about CLIP protein functions was observed in *Caenorhabditis elegans*. In *C. elegans*, the synergism between the CLASP2 orthologues CLS-2, BUB-1, and KLN-1, recruited by the kinetochore, seems to be crucial during the mitotic central spindle microtubule assembly [18,19]. Similarly, during *Xenopus laevi* embryogenesis, CLASPs, mainly transcribed in cranial nerves, cooperate with microtubule plus-end tracking proteins (+TIPs) in the regulation of microtubule dynamics [20,21]. In *Drosophila melanogaster*, the gene Orbit/Mast was identified as the orthologue of the mammalian CLASP2, and it was observed to be involved in the bundling of mitotic microtubules [22,23]. However, multiple studies showed that Orbit/Mast protein plays a key role in the meiotic division of germline cells [24]. In detail, Orbit/Mast is required at several stages of oogenesis, specifically during the formation of polarized 16-cell cysts before oocyte differentiation [25]. On the other hand, studies of immunofluorescence performed in male germline cells showed a very dynamic expression of Orbit/Mast, suggesting this protein plays a role in spermatogenesis. In spermatogonia, expression of Orbit was observed to be higher in the fusomes (germline-specific cytoskeleton), where it is involved in the proper orientation of spindles during synchronous mitosis of spermatogonium cysts [26]. During cytokinesis, the accumulation of myosin was seen to be dependent on Orbit, as the myosin heavy chain localizes in proximity to Orbit in the cleavage furrow [27,28]. However, Orbit/Mast seems to also participate in the premeiotic stage of male germ cells, as it was observed to be expressed during centriole elongation [29].

As it concerns the role of CLASP2 in fish models, at present, very scant literature is present. One comparative study in mammalian cardiomyocytes and zebrafish (*Danio rerio*) embryos showed that the formation of complex CLASP2-EB1 (microtubule end-binding protein 1), as it happens in human cells, is essential for cardiac sodium channel NaV1.5 trafficking, suggesting a similar scaffold role of this protein in microtubule stabilization [30]. Moreover, interesting insights have risen on CLASP2’s involvement in the regeneration processes concerning fish nervous and hemopoietic systems. Specifically, gene expression and pathway enrichment studies in the zebrafish spinal cord injury model underlined an upregulation of the CLASP2 gene associated with axon regeneration, as it acts on microtubules to favor axon extension [30,31]. Similarly, a comparative study in mice and zebrafish showed that CLASP2 deficiency affects Hematopoietic Stem Cells (HSCs) expansion and maturation during animal development as it regulates c-Kit protein levels and Golgi apparatus integrity [32]. These previous observations seem to confirm a key role of CLASP2 in fish mitotic cell division as well as in mammals and other vertebrate models. However, there are no studies that have clearly defined its role during fish germ cell formation.

In the present studies, we aim to analyze *clasp2* gene transcription levels during fish germ line cell formation. We decided to use two excellent fish models, zebrafish and guppy (*Poecilia reticulata*) teleost fishes, in which oogenesis and spermatogenesis processes are best described [33,34,35,36]. In detail, by quantitative PCR, we measured total *clasp2* transcription levels in different zebrafish and guppy tissues, observing a higher transcription in the testis, similarly in the two models. Then, we specifically localized *clasp2* mRNA by in situ hybridization, showing *clasp2* high transcription in spermatozoa in zebrafish and spermatozeugmata in guppy.

## 2. Materials and Methods

### 2.1. Phylogenetic Analysis

Sequences of the homology cluster of the *clasp* gene family were retrieved from the results of Piccinini and Milani (2023) [37] for a subset of metazoan species (namely, zebrafish, *P. reticulata*, other *Gnathostomata* species, *Drosophila melanogaster*, and *Caenorhabditis elegans*). Sequences were aligned with MAFFT [38], trimmed with BMGE [39], and a Maximum Likelihood tree was inferred with IQTREE2 [40]. *Amphimedon queenslandica* (Porifera) was used as an outgroup to root the tree.

### 2.2. Dissection of Animals and Organs

The study was performed by using *AB zebrafish (*Danio rerio*) and guppy (*Poecilia reticulata*) adult males and females, respectively, obtained from a local supplier and recently analyzed for other purposes, as described in our previous published reports [34,41]. Fish were housed in an aquarium under standard photoperiod conditions (14 h light and 10 h dark) and a temperature of 28 °C. The animals have not received any medical treatment before or during the experiments.

All procedures have been performed by Italian Government Decree 26/2014 and approved by the Animal Ethical Committee of the University of Bologna (protocol no. 17/79/2014). Euthanasia of animals has been performed by using specific anesthetic drugs (ethyl 3-aminobenzoate and methanesulfonate 0.1%, Sigma Chemicals Co., St. Louis, MO, USA), and organs have been dissected for the experimental procedures.

### 2.3. RNA Extraction

Total RNA was extracted from different organs (3 adult males and 3 adult females for zebrafish), and 3 adult males and 3 adult females for guppy were sacrificed as described above. The following organs, from different specimens: brains, eyes, kidneys, testis, and ovaries, were dissociated using RLT buffer with the RNAeasy mini kit (Qiagen, Frankfurt, Germany). To obtain purified RNA, we followed the manufacturer’s protocol. This procedure was repeated in three independent experiments.

### 2.4. Reverse Transcriptase PCR

Reverse transcription of mRNA into cDNA was obtained by using the Superscript III First-Strand Synthesis System kit (Invitrogen, Boston, MA, USA), according to the manufacturing procedure. In detail, 0.5 μg of total RNA was incubated with buffer and enzyme mix for 10 min at 25 °C, 30 min at 50 °C, and 5 min at 85 °C. After, samples were treated with RNase-H for 30 min at 37 °C and stored at −20 °C.

### 2.5. Quantitative Real-Time PCR

Quantitative real-time polymerase chain reaction (PCR) was performed by using the thermocycler with the MyiQ detector (Bio-Rad, Hercules, Dallas, TX, USA). Specifically, cDNA has been mixed with specific forward and reverse primers, SYBR-Green (Bio-Rad), and RNase-free water according to the manufacturer’s protocol. The above mix was incubated for 15 min at 95 °C, then 15 s at 95 °C, 30 s at 60 °C, and 30 s at 72 °C for 40 cycles. Primer sequences used for amplification of *clasp2* and *gapdh* (housekeeping) in zebrafish and guppy are listed in Table 1 and Table 2:

qPCR data indicate the fold change of *clasp2* transcript levels in the brain, eye, kidney, testis, and ovary of zebrafish and guppy, using *gapdh* to normalize the absolute quantification, calculated using 2−∆∆Ct. Correct amplification and PCR efficiency were confirmed by melting curve analysis. Each qPCR experiment has been performed in biological triplicates. In the analysis of qPCR data, each n represents the average of triplicates from a single experiment. Each experiment has been repeated three times.

### 2.6. Synthesis of Riboprobes for clasp2 in Zebrafish and Guppy

All digoxigenin (DIG)-labeled antisense and sense riboprobes were generated using the protocol described in our previous studies [42]. In detail, *clasp2* riboprobes for zebrafish and guppy were produced by using primers listed in Table 3 and Table 4.

After amplification by PCR, each insert was cloned into the TOPO-TA vector (Invitrogen). Subsequently, transformation into thermos-competent cells was performed. After applying heat shock, bacteria were plated onto Luria–Bertani (LB) agar plates containing the appropriate antibiotic to select only the transformed cells. The transformants were screened, and the white colonies containing the insert were then grown. Then, white bacterial colonies were picked and inoculated in an LB medium containing the appropriate antibiotic. Bacteria were grown for 16 h in an orbital rotator at 37 °C. Subsequently, purification of plasmid DNA was performed using the Quick Plasmid Miniprep Kit (Invitrogen, Boston, MA, USA). Next, we confirmed the antisense and sense orientation by sequencing, and the plasmids were linearized by the enzymatic restriction. Next, using T7 polymerase (Roche-Diagnostic) and SP6 polymerase (Roche-Diagnostic, Barrington, IL, USA) with DIG-RNA Labeling Mix (Roche Diagnostic, Indianapolis, IN, USA), we performed in vitro transcription. All riboprobes were purified using NucleoSpin RNA Clean-up columns (Qiagen, Frankfurt, Germany). Verification of reaction specificity was performed by hybridizing the sense and antisense riboprobes on adjacent sections.

### 2.7. In Situ Hybridization

Testis from zebrafish and guppy (*n* = 3 males per species) were quickly dissected and fixed in paraformaldehyde (PFA) at 4% overnight at 4 °C. After 24 h, tissues were processed for paraffin embedding. Sections (7 µm thick) were cut by using a rotary microtome and mounted on slides. All paraffin sections were immersed in xylene two times for 3 min and rehydrated in ethanol at 100%, 95%, 80%, 70%, 50%, and 30% (1 min each). Next, sections of zebrafish and guppy testis were immersed in PBS, adding proteinase K diluted at 2 mg/mL at 37 °C for 5 min. All slides were fixed in paraformaldehyde 4% for 20 min, washed in PBS, and then washed in standard saline sodium citrate (SSC) twice (10 min each). Then, slides were incubated at 63 °C for 24 h in a moist chamber with the probes (2 µg/mL) diluted in a specific medium (Denhart 5×; SSC 2×; 50% formamide; ethylenediamine-tetra acetic acid 4 mM; 5% dextran sulfate; yeast tRNA 50 µg/mL). After 24 h, slides were washed with SCC 2×; 50% formamide/SCC 2×; SSC 0.2×; and SSC 0.1×. After, sections were immersed in Tris-HCl/NaCl buffer (mixing 100 mM Tris-HCl pH 7.5 and 150 mM NaCl) and washed in the same buffer containing 0.5% milk powder and 0.1% Triton. For chromogenic revelation, all sections have been incubated with anti-digoxigenin alkaline phosphatase Fab fragments, dilution 1:5000 (Roche Diagnostic Company, Chicago, IL, USA), overnight at room temperature. After 24 h, all sections were washed in Tris-HCl/NaCl buffer and with 110 mM HCl-Tris (pH 8) containing 10 mM MgCl_2_ and 100 mM NaCl. Staining was performed using NBT/BCIP buffer (Roche, Mannheim, Germany) (pH 9.5). For fluorescence revelation, all slides were immersed in anti-DIG POD antibody (Roche, Germany) at a 1:200 dilution in the above blocking solution at room temperature for 24 h. Slides were washed for 5 min in PBS 4 times. Next, the sections were visualized by the HNPP/Fast Red Fluorescent Detection set (Roche Diagnostics, Chicago, IL, USA, #11758888001) according to the kit instructions. Mounted with DAPI (Thermo Fisher, Waltham, MA, USA) and cover slide and then observed with a fluorescence microscope (Olympus Life Science, Segrate, Milan, Italy), equipped with a DP71 digital camera, or a confocal microscope (Leica SP2). The images were processed with either the Olympus (Cell), Zeiss (AxioVision4) (Zeiss, Jana, Germany), or Leica (LCS Lite) (Leica, Wetzlar, Germania) software V-10. For all reagents and kits see Table 5.

### 2.8. Histology (Haematoxylin and Eosin Staining)

Adult zebrafish testes were fixed in 4% paraformaldehyde in PBS for 24h at 4 °C. After washing, the tissues were stored in 70% ethanol at 4 °C. Subsequently, tissues were embedded in paraffin, sectioned using a rotary microtome, and sections were mounted on slides. To deparaffinize, sections were put in xylene and rehydrated in an ethanol series. After, paraffin sections were washed 3× in distilled water for 1 min each time. Then, to examine cell and tissue morphology, hematoxylin and eosin (HE) staining was performed.

### 2.9. Statistical Analysis

Data were processed for statistical analysis using GraphPad Prism version 9.4.1 by applying one-way ANOVA with Tukey–Kramer post hoc tests, adjusted for multiple comparisons. Values of *p* equal to or less than 0.5 were considered statistically significant.

Catalog number for all kits and reagents used:

**Table 5:** Catalog number of kits and reagents.

RNAeasy minikit (Qiagen)	cat # 74104
Superscript III First-Strand Synthesis kit	cat # 12574026
TOPO™ TA Cloning™	cat # K4575J10
Dig-RNA label mix (Roche)	cat # 11277073910
HNPP/Fast Red Fluorescent Detection set	cat # 11758888001
Anti-Dig antibody (Roche)	cat # 11093274910
Proteinase K	cat # 25530049
Ethanol	cat # 100983

## 3. Results

### 3.1. Phylogenetic Tree of the Clasp Gene Family

**Figure 1:**
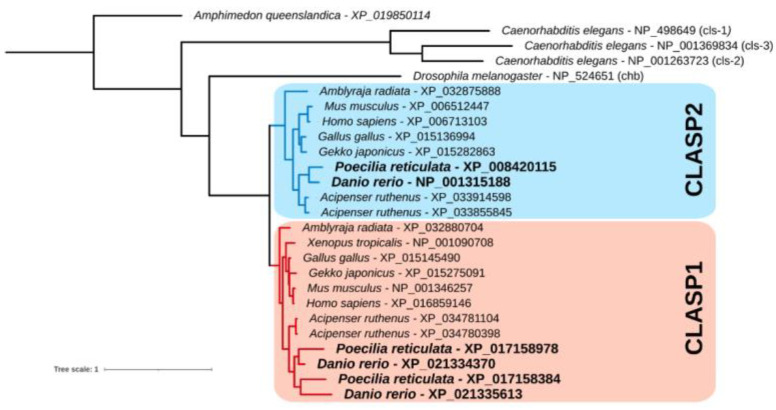
Phylogenetic tree of the *clasp* gene family.

The Maximum Likelihood tree showed that the emergence of the CLASP1 and CLASP2 paralogues preceded the evolution of gnathostomes but followed at least the emergence of deuterostomes. A subsequent duplication of CLASP1 occurred in the teleost lineage (Figure 1).

### 3.2. Quantitative Analysis of clasp2 Transcription Level in Different Organs of Zebrafish and Guppy

**Figure 2:**
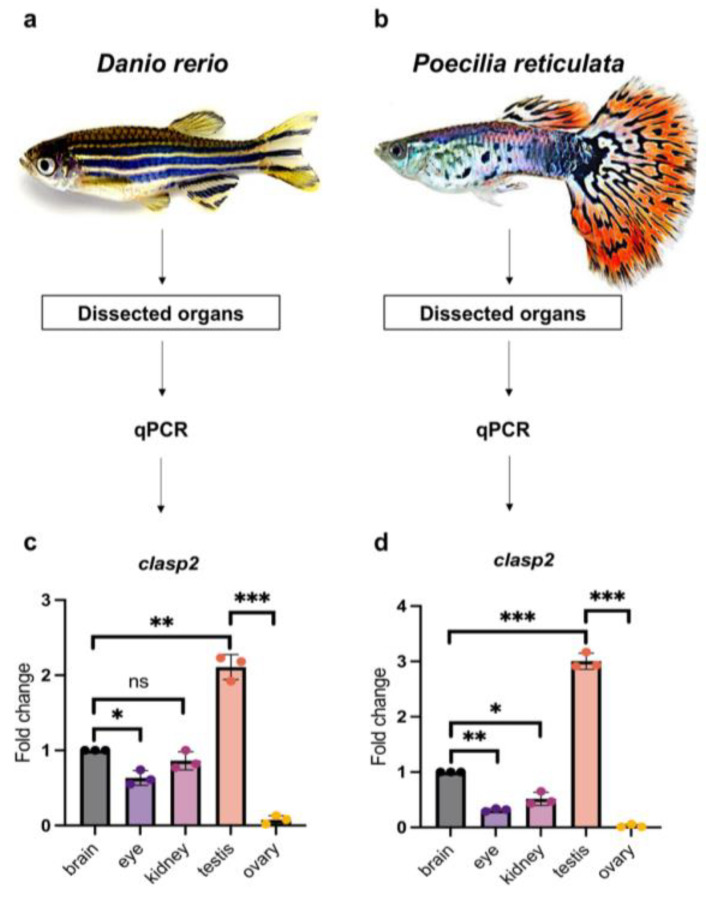
(**a**) Schematic experimental design outline for organs dissected from adult zebrafish (*Danio rerio*). (**b**) Schematic experimental design outline for organs dissected from an adult guppy (*Poecilia reticulata*). (**c**) qPCR analysis of *clasp2* in the brain, eye, kidney, testis, and ovary of adult zebrafish. *Clasp2* is most transcribed in the testis. Statistical analysis was completed using a one-way ANOVA with Tukey–Kramer post hoc tests, adjusted for multiple comparisons (*n* = 3 animals used). * *p* < 0.01; ** *p* < 0.001; *** *p* < 0.0001. ns—not significant. (**d**) qPCR analysis of *clasp2* in the brain, eye, kidney, testis, and ovary of an adult guppy. *Clasp2* is most transcribed in the testis. Statistical analysis was completed using a one-way ANOVA with Tukey–Kramer post hoc tests, adjusted for multiple comparisons (*n* = 3 animals used). * *p* < 0.01; ** *p* < 0.001; *** *p* < 0.0001.

For qPCR experiments, firstly, we dissected adult males and females of zebrafish (*Danio rerio*) and guppy (*Poecilia reticulata*) to remove different organs from each teleost fish (Figure 2a,b). Next, we measured the transcription level of *clasp2* in the brain, eye, kidney, testis, and ovary.

In the ovary of zebrafish and guppy, *clasp2* was undetected. In the kidney and eye of both teleost fish, *clasp2* presented lower transcription compared with the brain. The most relevant data obtained from this analysis is that *clasp2* is highly transcribed in the testis of adult zebrafish and guppy compared with other organs (Figure 2c,d).

### 3.3. Clasp2 Transcript Is Transcribed in Spermatozoa of Adult Zebrafish Testis

We first performed hematoxylin-eosin staining of adult zebrafish testis sections to better show germ line cell morphology. In zebrafish, it is well described that spermatogenesis occurs in cysts [43]. As shown in Supplementary Figure S1, we observed numerous cysts, characterized by spermatogonia displaying heterochromatin in elongated and/or round nuclei. In the figure, it is possible to identify spermatocytes (at different meiotic division stages). Instead, spermatids, and finally, spermatozoa, are identifiable by the significant decrease in cellular and nuclear volumes.

**Figure 3:**
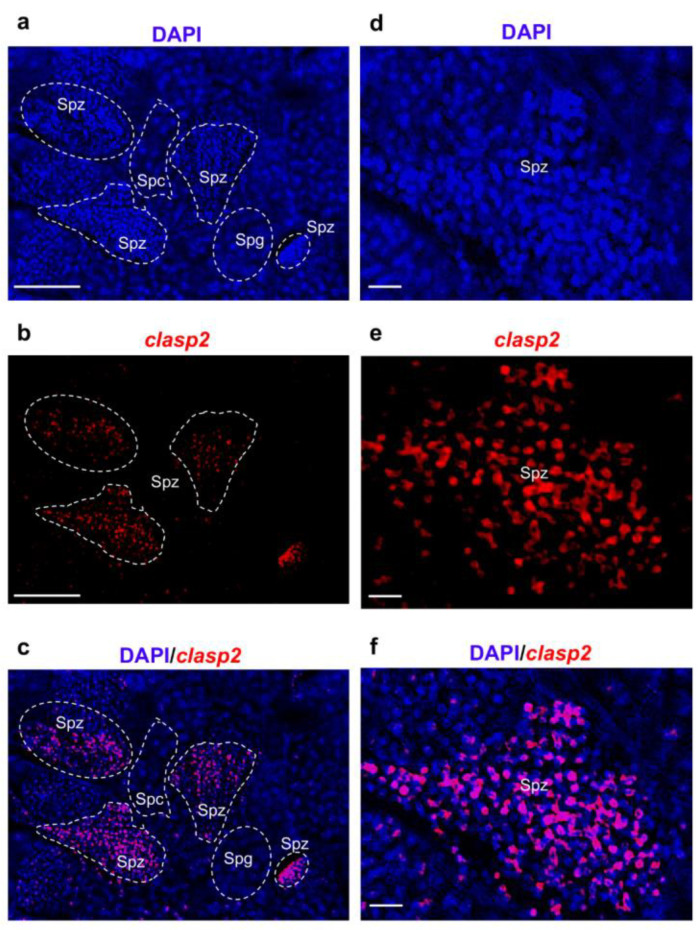
*Clasp2* mRNA transcription pattern in adult zebrafish testis at widefield fluorescence microscopy. (**a**) DAPI staining marks cell nuclei. (**b**) Fluorescence in situ hybridization of *clasp2* in adult zebrafish testis. (**c**) Fluorescence in situ hybridization of *clasp2* and cell nuclei (DAPI). (**d**) High magnification of spermatozoa cell nuclei (DAPI). (**e**) High magnification of *clasp2* expressing cells. (**f**) High magnification of fluorescence in situ hybridization of *clasp2* and cell nuclei (DAPI). Abbreviations: Spz: spermatozoa; Spc: spermatocytes; Spg: spermatogonia (inside the dashed line) in adult zebrafish testis. Scale bars: 100 µ (**a**–**c**) and 25 µ (**d**–**f**).

Subsequently, to identify *clasp2* expressing cells in adult zebrafish testis, we performed fluorescence in situ hybridization. *Clasp2* mRNA was specifically transcribed in spermatozoa (Figure 3a–f).

**Figure 4:**
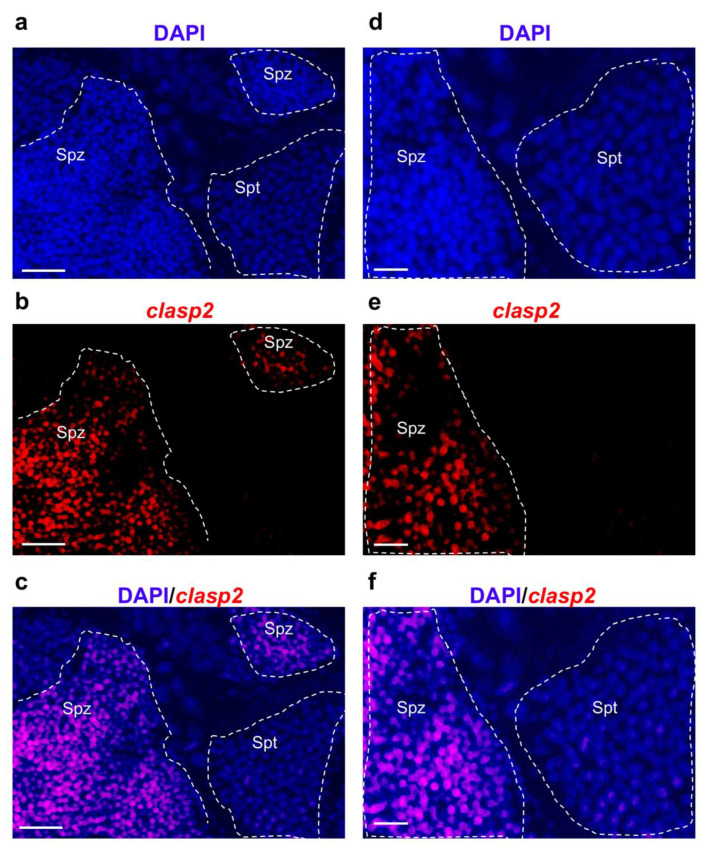
*Clasp2* transcription pattern in adult zebrafish testis at confocal microscopy. (**a**) DAPI staining marks cell nuclei. (**b**) Fluorescence in situ hybridization of *clasp2* in adult zebrafish testis. (**c**) Fluorescence in situ hybridization of *clasp2* and cell nuclei (DAPI) in adult zebrafish testis. (**d**) DAPI staining marks cell nuclei at high magnification. (**e**) Fluorescence in situ hybridization of *clasp2* in adult zebrafish testis at high magnification. (**f**) Fluorescence in situ hybridization of *clasp2* and cell nuclei (DAPI) in adult zebrafish testis at high magnification. Abbreviations: Spz; spermatozoa. Spt; spermatids. Scale bars: 100 µ (**a**–**c**) and 25 µ (**d**–**f**).

We confirmed previous data by using confocal microscopy with higher magnification (Figure 4a–f).

### 3.4. Clasp2 Transcript Is Transcribed in Spermatozeugmata of Adult Guppy Testis

**Figure 5:**
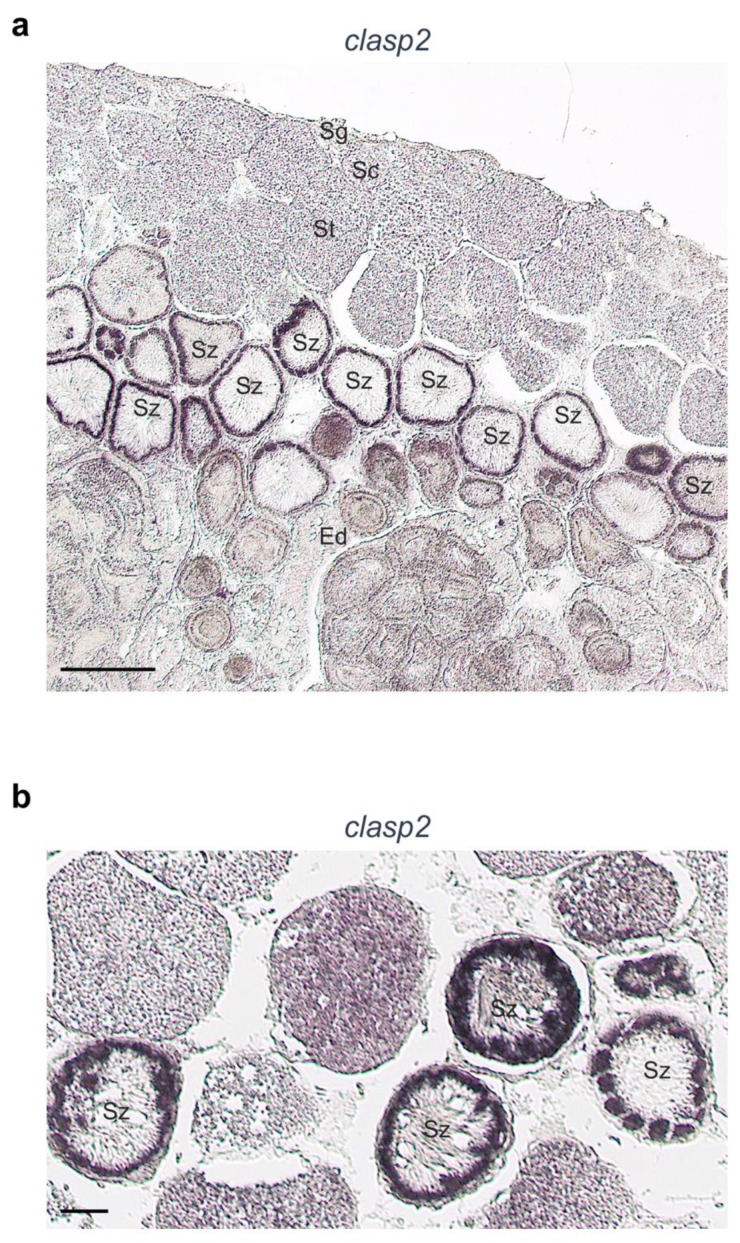
*Clasp2* transcript distribution in sections of adult guppy testis. (**a**) In this section, we identified the organization of the testis. Cysts containing germ cells in the early stages of spermatogenesis (spermatogonia and spermatocytes) are located near the periphery of the testis, while cysts with numerous spermatids at different stages of spermiogenesis are located deeper, near the efferent ducts. In the same region, there are present spermatozeugmata cysts, in which spermatozoa are tightly packed with the sperm heads oriented towards the Sertoli cells and flagella oriented towards the center of the cyst. Spermatozeugmata is also found inside the efferent ducts (located in the central region of the testis), where mature spermatozoa are released. Chromogenic in situ hybridization of *clasp2* in this section shows a clear marking at the periphery of the spermatozeugmata. The peripheral region of spermatozeugmata cysts located inside the efferent ducts shows weak staining (Sg: spermatogonia; Sc: spermatocytes; St: spermatids; Sz: spermatozeugmata; Ed: efferent ducts). (**b**) The strong marking of spermatozeugmata. (Sz: spermatozeugmata). Scale bars: 100 µ (**a**); 50 µ (**b**).

As we mentioned before, the present study aimed to compare *clasp2* mRNA transcription patterns between zebrafish and guppy testis. Therefore, to identify the distribution of *clasp2* transcripts in guppy testis, we performed chromogenic and fluorescence in situ hybridization. By chromogenic revelation, we found that the *clasp2* transcript was specifically transcribed in spermatozeugmata. In detail, the staining results are dashed and present only at the periphery of spermatozeugmata where the spermatozoa heads make contact with the Sertoli cells (Figure 5a,b).

**Figure 6:**
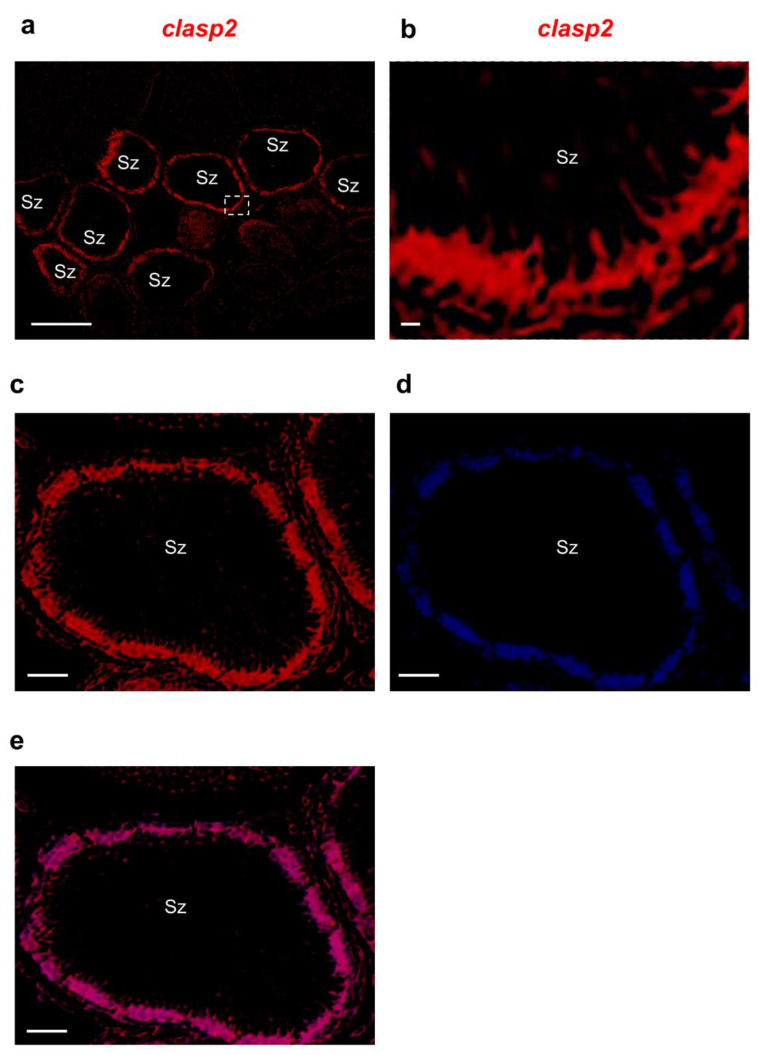
*Clasp2* mRNA distribution in adult guppy testis, confocal microscopy. (**a**) Fluorescence in situ hybridization of *clasp2* shows the labeling concentrated at the periphery of numerous spermatozeugmata. (**b**) High magnification of a portion of spermatozeugmata in a white rectangle is shown in panel a. (**c**) Fluorescence in situ hybridization of *clasp2* is seen at the periphery of spermatozeugmata cysts. (**d**) DAPI staining marks all the cell nuclei of the testis. (**e**) The merge of fluorescence in situ hybridization of *clasp2* and DAPI in adult guppy testis. Abbreviation: Sz; spermatozeugmata. Scale bars are 50 µ (**a**); 10 µ (**b**); and 30 µ (**c**–**e**).

We confirmed these previous results by using fluorescence in situ hybridization. We found that *clasp2* transcripts are specifically distributed in spermatozoa contained in spermatozeugmata cysts (Figure 6a–e).

## 4. Discussion

Microtubule dynamics and function during spindle assembly in meiotic cell divisions are crucial for the correct development of both somatic and germ cell lines in most animal species. The CLASPs protein family is a highly important regulator of the growing microtubule plus-ends, as indicated by their enrichment at these sites via tip-binding proteins such as end-binding proteins [44] (EB). Microtubule ends are also regulated by localized multimolecular ensembles of CLASPs [16,45]. For example, membrane-associated clusters of CLASP molecules capture microtubule ends at the postsynaptic membrane, whereas cortical clusters of CLASPs tether and stabilize microtubule ends near focal adhesions in motile cells or at the cell cortex [46]. Despite several animal models, such as *Drosophila*, *Xenopus*, and *C. elegans*, CLASP2 activity in meiotic divisions has been well described, and very scant knowledge is present for fish models.

For the first time in the present study, we showed the transcription pattern of *clasp2* during spermatogenesis in two small teleost fish, zebrafish and guppy. We found that *clasp2* is transcribed in several tissues, such as the brain, eye, and kidney, in a comparable fashion between zebrafish and guppy; however, higher transcription was observed in the testis. These data agree with what has been previously observed in other animal models, such as *Mus musculus* or *Drosophila* [47,48]. Thus, this might suggest a similar potential role in male germ cell differentiation. In detail, by in situ hybridization in zebrafish, we found that *clasp2* is specifically transcribed in the spermatozoa of adult testes. Interestingly, in the guppy, *clasp2* is highly transcribed in spermatozeugmata. Therefore, we could hypothesize that *clasp2* plays an important role in male germ cells during the late stage of spermatogenesis (i.e., spermiogenesis). Indeed, our results confirm previous studies performed using mouse models. In detail, breedings between homozygous CLASP2 knockout mice did not yield any pregnancies; hence, separate breedings between homozygous males and heterozygous or wild-type females did not produce any litter. This evidence led the authors to hypothesize that both male and female CLASP2^−/−^ mice might be infertile. Further examination of the ovaries showed no pronounced difference in size but a difference in coloration due to hemorrhages. By contrast, testes from CLASP2 knockout mice were severely reduced in size in comparison to wild-type testes. Further histological analysis of the testes of CLASP2 knockout mice showed absent mature sperm, and the interstitium of the tubule was increased in size and filled with blood vessels [48]. In humans as well as in other animals, it is well established that spermatogenesis relies on dynamic changes in the cytoskeleton, which implies a strict regulation of microtubule dynamics [49]. Thus, microtubule dysfunction can alter the correct formation of male germ cells, and it is associated with infertility [50]. Interestingly, one recent study observed that ablation of the CLIP-170 protein (belonging to the same family of proteins as CLASP2) is associated with abnormal nucleus shapes of spermatozoa and consequent infertility [51]. However, to the best of our knowledge, no evidence has been reported about the specific role of CLASP2 in human male spermatogenesis. A possible speculation about the role of CLASP2 in male fertility could derive from preliminary data reported on The Human Protein Atlas on CLASP2 expression in Testis Cancer (https://www.proteinatlas.org/ENSG00000163539-CLASP2/pathology/testis+cancer (accessed on 12 October 2023). Indeed, it is well recognized that testicular cancer exerts profound detrimental effects on the reproductive health of men [52,53]. However, the data reported on the Protein Atlas are still preliminary, and, at present, they have not reached any prognostic value.

Thus, despite more extensive studies being needed to decipher whether and how CLASP2 is involved in fish spermatogenesis, our study represents the very first evidence of *clasp2* expression in developing male germ line cells, giving insight to further investigate this protein as a target in teleost fish meiotic cell division.

## Figures and Tables

**Table 1:** qPCR forward and reverse primer sequences of *clasp2* and *gapdh* in zebrafish.

	Zebrafish	(Amplicon Size)
*clasp2*	F: TGGAGGCACATAAAGACC	(122 bp)
	R: TGACTGGATGATGGGACA	
*Gapdh*	F: GTGTAGGCGTGGACTGTGG	(151 bp)
	R: TGGGAGTCAACCAGGACAAA	

**Table 2:** qPCR forward and reverse primer sequences of *clasp2* and *gapdh* in guppy.

	Guppy	(Amplicon Size)
*clasp2*	F: GAAGGACGTTACACGTAGAC	(181 bp)
	R: CCCACAGATGTCTATCCCT	
*Gapdh*	F: CTCCACTCATGGTGTCTG	(140 bp)
	R: CAACATAGTCTACGGCAGC	

**Table 3:** Forward and reverse primer sequences for *clasp2* riboprobes in zebrafish.

	Zebrafish	Length of Riboprobe
*clasp2*	F: GCATTGCTGGGGATCGATA	(532 bp)
	R: CGTCGAAACTACGGTCTTG	

**Table 4:** Forward and reverse primer sequences for *clasp2* riboprobes in guppy.

	Guppy	Length of Riboprobe
*clasp2*	F: CTCAGCTCAGGCTGCTTT	(494 bp)
	R: CGCAGTTGGGAATGAGGT	

## Data Availability

All data generated in this study are available from the corresponding author on reasonable request.

## Supplementary Materials

The following supporting information can be downloaded at: https://www.mdpi.com/article/10.3390/ani13233617/s1, Supplementary Figure S1: Hematoxylin-eosin staining of adult zebrafish testis. Spg: spermatogony. Spc: spermatocyte. Spt: spermatid. Spz: spermatozoa.

## References

[1] Drabek K., van Ham M., Stepanova T., Draegestein K., van Horssen R., Sayas C.L., Akhmanova A., Ten Hagen T., Smits R., Fodde R. (2006). Role of CLASP2 in microtubule stabilization and the regulation of persistent motility. Curr. Biol..

[2] Al-Bassam J., Chang F. (2011). Regulation of microtubule dynamics by TOG-domain proteins XMAP215/Dis1 and CLASP. Trends Cell Biol..

[3] Lawrence E.J., Arpag G., Norris S.R., Zanic M. (2018). Human CLASP2 specifically regulates microtubule catastrophe and rescue. Mol. Biol. Cell.

[4] Pereira A.L., Pereira A.J., Maia A.R., Drabek K., Sayas C.L., Hergert P.J., Lince-Faria M., Matos I., Duque C., Stepanova T. (2006). Mammalian CLASP1 and CLASP2 cooperate to ensure mitotic fidelity by regulating spindle and kinetochore function. Mol. Biol. Cell.

[5] Matsui T., Watanabe T., Matsuzawa K., Kakeno M., Okumura N., Sugiyama I., Itoh N., Kaibuchi K. (2015). PAR3 and aPKC regulate Golgi organization through CLASP2 phosphorylation to generate cell polarity. Mol. Biol. Cell.

[6] Adachi A., Kano F., Tsuboi T., Fujita M., Maeda Y., Murata M. (2010). Golgi-associated GSK3beta regulates the sorting process of post-Golgi membrane trafficking. J. Cell Sci..

[7] Dillon G.M., Tyler W.A., Omuro K.C., Kambouris J., Tyminski C., Henry S., Haydar T.F., Beffert U., Ho A. (2017). CLASP2 Links Reelin to the Cytoskeleton during Neocortical Development. Neuron.

[8] Sayas C.L., Basu S., van der Reijden M., Bustos-Moran E., Liz M., Sousa M., Van Ijcken W.F., Avila J., Galjart N. (2019). Distinct Functions for Mammalian CLASP1 and -2 During Neurite and Axon Elongation. Front. Cell Neurosci..

[9] Zhu B., Qi L., Liu S., Liu W., Ou Z., Chen M., Liu L., Zu X., Wang J., Li Y. (2017). CLASP2 is involved in the EMT and early progression after transurethral resection of the bladder tumor. BMC Cancer.

[10] Chen L., Xiong W., Guo W., Su S., Qi L., Zhu B., Mo M., Jiang H., Li Y. (2019). Significance of CLASP2 expression in prognosis for muscle-invasive bladder cancer patients: A propensity score-based analysis. Urol. Oncol..

[11] Valter A., Luhari L., Pisarev H., Truumees B., Planken A., Smolander O.P., Oselin K. (2023). Genomic alterations as independent prognostic factors to predict the type of lung cancer recurrence. Gene.

[12] Beffert U., Dillon G.M., Sullivan J.M., Stuart C.E., Gilbert J.P., Kambouris J.A., Ho A. (2012). Microtubule plus-end tracking protein CLASP2 regulates neuronal polarity and synaptic function. J. Neurosci..

[13] Siino V., Amato A., Di Salvo F., Caldara G.F., Filogamo M., James P., Vasto S. (2018). Impact of diet-induced obesity on the mouse brain phosphoproteome. J. Nutr. Biochem..

[14] Langlais P., Dillon J.L., Mengos A., Baluch D.P., Ardebili R., Miranda D.N., Xie X., Heckmann B.L., Liu J., Mandarino L.J. (2012). Identification of a role for CLASP2 in insulin action. J. Biol. Chem..

[15] Kruse R., Krantz J., Barker N., Coletta R.L., Rafikov R., Luo M., Hojlund K., Mandarino L.J., Langlais P.R. (2017). Characterization of the CLASP2 Protein Interaction Network Identifies SOGA1 as a Microtubule-Associated Protein. Mol. Cell Proteomics.

[16] Karki P., Ke Y., Zhang C.O., Li Y., Tian Y., Son S., Yoshimura A., Kaibuchi K., Birukov K.G., Birukova A.A. (2021). SOCS3-microtubule interaction via CLIP-170 and CLASP2 is critical for modulation of endothelial inflammation and lung injury. J. Biol. Chem..

[17] Karki P., Ke Y., Tian Y., Ohmura T., Sitikov A., Sarich N., Montgomery C.P., Birukova A.A. (2019). Staphylococcus aureus-induced endothelial permeability and inflammation are mediated by microtubule destabilization. J. Biol. Chem..

[18] Maton G., Edwards F., Lacroix B., Stefanutti M., Laband K., Lieury T., Kim T., Espeut J., Canman J.C., Dumont J. (2015). Kinetochore components are required for central spindle assembly. Nat. Cell Biol..

[19] Nahaboo W., Zouak M., Askjaer P., Delattre M. (2015). Chromatids segregate without centrosomes during Caenorhabditis elegans mitosis in a Ran- and CLASP-dependent manner. Mol. Biol. Cell.

[20] Park E.C., Lee H., Hong Y., Kim M.J., Lee Z.W., Kim S.I., Kim S., Kim G.H., Han J.K. (2012). Analysis of the expression of microtubule plus-end tracking proteins (+TIPs) during Xenopus laevis embryogenesis. Gene Expr. Patterns.

[21] Grimaldi A.D., Zanic M., Kaverina I. (2015). Encoding the microtubule structure: Allosteric interactions between the microtubule +TIP complex master regulators and TOG-domain proteins. Cell Cycle.

[22] Akhmanova A., Hoogenraad C.C., Drabek K., Stepanova T., Dortland B., Verkerk T., Vermeulen W., Burgering B.M., De Zeeuw C.I., Grosveld F. (2001). Clasps are CLIP-115 and -170 associating proteins involved in the regional regulation of microtubule dynamics in motile fibroblasts. Cell.

[23] Aonuma M., Miyamoto M., Inoue Y.H., Tamai K., Sakai H., Kamasawa N., Matsukage A. (2005). Microtubule bundle formation and cell death induced by the human CLASP/Orbit N-terminal fragment. Cell Struct. Funct..

[24] Moriwaki T., Goshima G. (2016). Five factors can reconstitute all three phases of microtubule polymerization dynamics. J. Cell Biol..

[25] Mathe E., Inoue Y.H., Palframan W., Brown G., Glover D.M. (2003). Orbit/Mast, the CLASP orthologue of Drosophila, is required for asymmetric stem cell and cystocyte divisions and development of the polarised microtubule network that interconnects oocyte and nurse cells during oogenesis. Development.

[26] Miyauchi C., Kitazawa D., Ando I., Hayashi D., Inoue Y.H. (2013). Orbit/CLASP is required for germline cyst formation through its developmental control of fusomes and ring canals in Drosophila males. PLoS ONE.

[27] Inoue Y.H., Savoian M.S., Suzuki T., Mathe E., Yamamoto M.T., Glover D.M. (2004). Mutations in orbit/mast reveal that the central spindle is comprised of two microtubule populations, those that initiate cleavage and those that propagate furrow ingression. J. Cell Biol..

[28] Kitazawa D., Matsuo T., Kaizuka K., Miyauchi C., Hayashi D., Inoue Y.H. (2014). Orbit/CLASP is required for myosin accumulation at the cleavage furrow in Drosophila male meiosis. PLoS ONE.

[29] Shoda T., Yamazoe K., Tanaka Y., Asano Y., Inoue Y.H. (2021). Orbit/CLASP determines centriole length by antagonising Klp10A in Drosophila spermatocytes. J. Cell Sci..

[30] Marchal G.A., Jouni M., Chiang D.Y., Perez-Hernandez M., Podliesna S., Yu N., Casini S., Potet F., Veerman C.C., Klerk M. (2021). Targeting the Microtubule EB1-CLASP2 Complex Modulates Na(V)1.5 at Intercalated Discs. Circ. Res..

[31] Shen W.Y., Fu X.H., Cai J., Li W.C., Fan B.Y., Pang Y.L., Zhao C.X., Abula M., Kong X.H., Yao X. (2022). Identification of key genes involved in recovery from spinal cord injury in adult zebrafish. Neural Regen. Res..

[32] Klaus A., Clapes T., Yvernogeau L., Basu S., Weijts B., Maas J., Smal I., Galjart N., Robin C. (2022). CLASP2 safeguards hematopoietic stem cell properties during mouse and fish development. Cell Rep..

[33] Leal M.C., Cardoso E.R., Nobrega R.H., Batlouni S.R., Bogerd J., Franca L.R., Schulz R.W. (2009). Histological and stereological evaluation of zebrafish (*Danio rerio*) spermatogenesis with an emphasis on spermatogonial generations. Biol. Reprod..

[34] Cacialli P. (2022). Expression of Nerve Growth Factor and Its Receptor TrkA in the Reproductive System of Adult Zebrafish. Vet. Sci..

[35] Cacialli P., D’Angelo L., de Girolamo P., Avallone L., Lucini C., Pellegrini E., Castaldo L. (2018). Morpho-Functional Features of the Gonads of Danio rerio: The Role of Brain-Derived Neurotrophic Factor. Anat. Rec..

[36] Torres-Martinez A., Ruiz de Dios L., Hernandez-Franyutti A., Uribe M.C., Sanchez W.C. (2019). Structure of the testis and spermatogenesis of the viviparous teleost *Poecilia mexicana* (Poeciliidae) from an active sulfur spring cave in Southern Mexico. J. Morphol..

[37] Piccinini G., Milani L. (2023). Germline-related molecular phenotype in Metazoa: Conservation and innovation highlighted by comparative transcriptomics. Evodevo.

[38] Katoh K., Standley D.M. (2013). MAFFT multiple sequence alignment software version 7: Improvements in performance and usability. Mol. Biol. Evol..

[39] Criscuolo A., Gribaldo S. (2010). BMGE (Block Mapping and Gathering with Entropy): A new software for selection of phylogenetic informative regions from multiple sequence alignments. BMC Evol. Biol..

[40] Minh B.Q., Schmidt H.A., Chernomor O., Schrempf D., Woodhams M.D., von Haeseler A., Lanfear R. (2020). IQ-TREE 2: New Models and Efficient Methods for Phylogenetic Inference in the Genomic Era. Mol. Biol. Evol..

[41] Milani L., Cinelli F., Iannello M., Lazzari M., Franceschini V., Maurizii M.G. (2022). Immunolocalization of Vasa, PIWI, and TDRKH proteins in male germ cells during spermatogenesis of the teleost fish *Poecilia reticulata*. Acta Histochem..

[42] Mahony C.B., Cacialli P., Pasche C., Monteiro R., Savvides S.N., Bertrand J.Y. (2021). Hapln1b, a central organizer of the ECM, modulates kit signaling to control developmental hematopoiesis in zebrafish. Blood Adv..

[43] Menke A.L., Spitsbergen J.M., Wolterbeek A.P., Woutersen R.A. (2011). Normal anatomy and histology of the adult zebrafish. Toxicol. Pathol..

[44] Girao H., Okada N., Rodrigues T.A., Silva A.O., Figueiredo A.C., Garcia Z., Moutinho-Santos T., Hayashi I., Azevedo J.E., Macedo-Ribeiro S. (2020). CLASP2 binding to curved microtubule tips promotes flux and stabilizes kinetochore attachments. J. Cell Biol..

[45] Rodgers N.C., Lawrence E.J., Sawant A.V., Efimova N., Gonzalez-Vasquez G., Hickman T.T., Kaverina I., Zanic M. (2023). CLASP2 facilitates dynamic actin filament organization along the microtubule lattice. Mol. Biol. Cell.

[46] Mimori-Kiyosue Y., Grigoriev I., Lansbergen G., Sasaki H., Matsui C., Severin F., Galjart N., Grosveld F., Vorobjev I., Tsukita S. (2005). CLASP1 and CLASP2 bind to EB1 and regulate microtubule plus-end dynamics at the cell cortex. J. Cell Biol..

[47] Sousa A., Reis R., Sampaio P., Sunkel C.E. (2007). The Drosophila CLASP homologue, Mast/Orbit regulates the dynamic behaviour of interphase microtubules by promoting the pause state. Cell Motil. Cytoskeleton.

[48] Drabek K., Gutierrez L., Vermeij M., Clapes T., Patel S.R., Boisset J.C., van Haren J., Pereira A.L., Liu Z., Akinci U. (2012). The microtubule plus-end tracking protein CLASP2 is required for hematopoiesis and hematopoietic stem cell maintenance. Cell Rep..

[49] O’Donnell L., O’Bryan M.K. (2014). Microtubules and spermatogenesis. Semin. Cell Dev. Biol..

[50] Gunes S., Sengupta P., Henkel R., Alguraigari A., Sinigaglia M.M., Kayal M., Joumah A., Agarwal A. (2020). Microtubular Dysfunction and Male Infertility. World J. Mens. Health.

[51] Lehti M.S., Sironen A. (2016). Formation and function of the manchette and flagellum during spermatogenesis. Reproduction.

[52] Paduch D.A. (2006). Testicular cancer and male infertility. Curr. Opin. Urol..

[53] Ostrowski K.A., Walsh T.J. (2015). Infertility with Testicular Cancer. Urol. Clin. N. Am..

