# Supplementary material for: Analysis of clasp2 Transcription Pattern in Male Germ Cells during Spermatogenesis: A Comparative Study in Zebrafish (Danio rerio) and Guppy (Poecilia reticulata)
Source: Animals (Basel). 2023 Nov 22;13(23):3617. doi: 10.3390/ani13233617 (PMC10705728; doi:10.3390/ani13233617)
Supplement: Supplementary file 1 [file animals-13-03617-s001.zip › animals-2688189-supplementary.pdf]

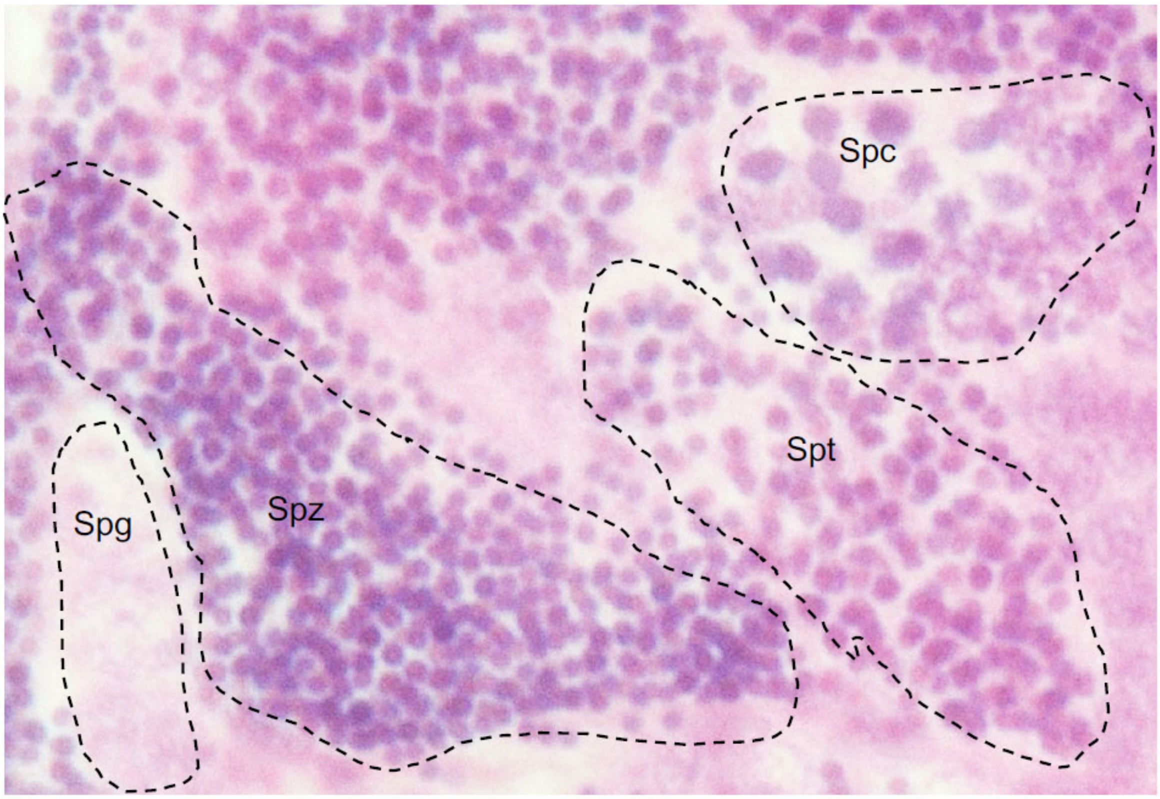

Supplementary Figure S1

Hematoxylin-eosin staining of adult zebrafish testis. Spg: spermatogony. Spc: spermatocyte. Spt: spermatid. Spz: spermatozoa
